# Mitigating Interfacial Contamination for Scalable Integration of Graphene in Neuroelectronic Devices

**DOI:** 10.1021/accountsmr.5c00259

**Published:** 2026-02-20

**Authors:** Aina Galceran, Marta Delgà-Fernández, Xavi Illa, Anton Guimerà-Brunet, Jose A. Garrido, Elena del Corro

**Affiliations:** † 231882 Catalan Institute of Nanoscience and Nanotechnology (ICN2), CSIC and BIST, 08193 Bellaterra, Spain; ‡ Institut de Microelectrònica de Barcelona (IMB-CNM), CSIC, Esfera UAB, 08193 Bellaterra, Spain; § Biomedical Research Networking Center in Bioengineering, Biomaterials and Nanomedicine (CIBER-BBN), 08028 Barcelona, Spain; ∥ ICREA, 08010 Barcelona, Spain

## Abstract

In the past decade, graphene has gained increasing attention as a material for the next generation of neuroelectronic interfaces thanks to its unique combination of properties, including transparency, flexibility, biocompatibility, and electrical performance. When integrated into thin-film technology microfabrication processes, graphene enables highly conformable and low invasive arrays of solution-gated field-effect transistors (gSGFETs), which are micrometric transducers that combine higher spatial density with the capability to record DC-coupled wide-bandwidth neural signals.

The same atomic scale nature of graphene that confers its exceptional sensitivity to surface charges also renders device performance strongly dependent on interfacial physicochemical phenomena at the substrate–graphene–medium boundary, which, in turn, are strongly dependent on the quality and pristine condition of the graphene layer. From a materials science perspective, maintaining the structural and electronic integrity of graphene throughout the entire microfabrication process represents a great challenge. Photolithographic processing often introduces polymeric residues, resulting in adsorbed charges and defect sites that induce residual doping, degrade mobility, and lead to time-dependent shifts in the transfer characteristics of gSGFETs. These changes induced by processing reduce sensor sensitivity and generate device-to-device variability, which currently limits reproducible benchmarking and scale-up of the technology.

In this Account, we highlight the remarkable potential of gSGFETs in neurotechnology and review the implications that the uncontrolled graphene surface states have on device behavior. We then present process engineering efforts aimed at addressing this issue through graphene cleaning methods. However, these cleaning approaches are necessarily mild to avoid damaging graphene and have not yet demonstrated the ability to fully ensure the homogeneity and reproducibility required for reliable technology. Finally, we examine emerging strategies based on the development of sacrificial protective layers, which act as effective barriers against process-induced contamination. We propose Cu as a particularly promising candidate given that, as the most common substrate for graphene growth, its etching chemistry has already been widely explored. The integration of sacrificial layers in the standardized microfabrication workflows represents a significant opportunity to improve the graphene-based technology’s reliability, enabling its advancement and translation toward industrial applications.

## Graphical abstract

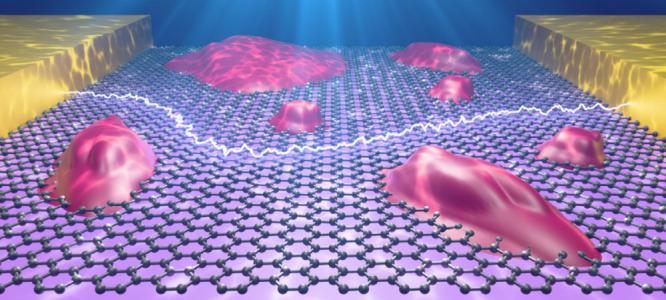

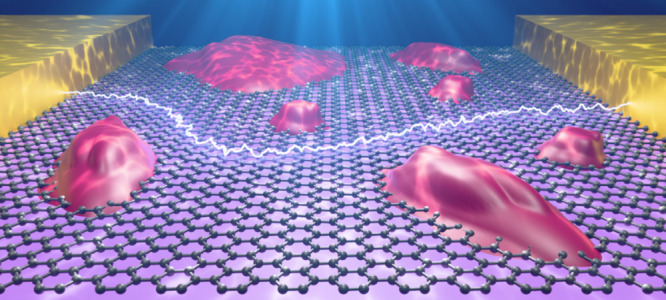

## 1 Graphene for Neurotechnology

Graphene possesses a unique set of physicochemical properties that make it an attractive candidate for bioelectronic and neurotechnology applications. As a one-atom-thick material, it presents an extreme surface-to-volume ratio, enabling efficient interactions with biological environments. Its very high carrier mobility renders graphene an ideal active channel material for solution-gated field-effect transistors (SGFETs), while its chemical stability and biocompatibility allow for operation in direct contact with the aqueous biological environment. Together, these features translate into a large interfacial capacitance and high signal sensitivity, properties that are particularly advantageous for bioelectronics and neural interfacing.

Graphene-based SGFET arrays (gSGFETs) have already demonstrated their potential as brain signal transducers for neural signals, converting extracellular electrophysiological activity into measurable current signals. Initial demonstrations of their performance were achieved in vitro with electrogenic cell cultures, establishing their ability to detect neuronal activity with high fidelity.^1^ Leveraging the intrinsic mechanical flexibility of graphene, gSGFETs can be integrated into microfabricated thin-film architectures to create flexible and conformal neural probes.^2^ Such devices can adapt to the brain’s soft and curve morphology, thereby improving signal quality while reducing tissue damage and inflammatory responses, which are critical for achieving long-term implantation and chronic recordings. Relative to more established neuroelectronic technologies, graphene field-effect transistors overcome the mechanical constrains of silicon-based devices and also outperform organic flexible transistors, exhibiting markedly enhanced performance and long-term stability.^3^

Beyond their mechanical advantages, gSGFETs operate as active transducers, providing intrinsic local amplification and reduced susceptibility to external noise sources compared to conventional passive electrodes.^4^ Their operating principle is depicted in Figure 1a.^5^ Briefly, the graphene channel current (measured between the drain and source terminals) is modulated by a gate potential applied through a reference electrode in the electrolyte. Electrical activity of the neural tissue in contact with the graphene surface modulates this channel current, which is subsequently converted into voltage signals via the transfer characteristics of the graphene transistor. Importantly, the transistor configuration not only enhances recording fidelity but also facilitates scalable architectures, as multiplexing schemes can be implemented to reduce the connectivity complexity and enable the development of high-density, addressable arrays for large-scale brain mapping.^6–8^

**Figure 1:**
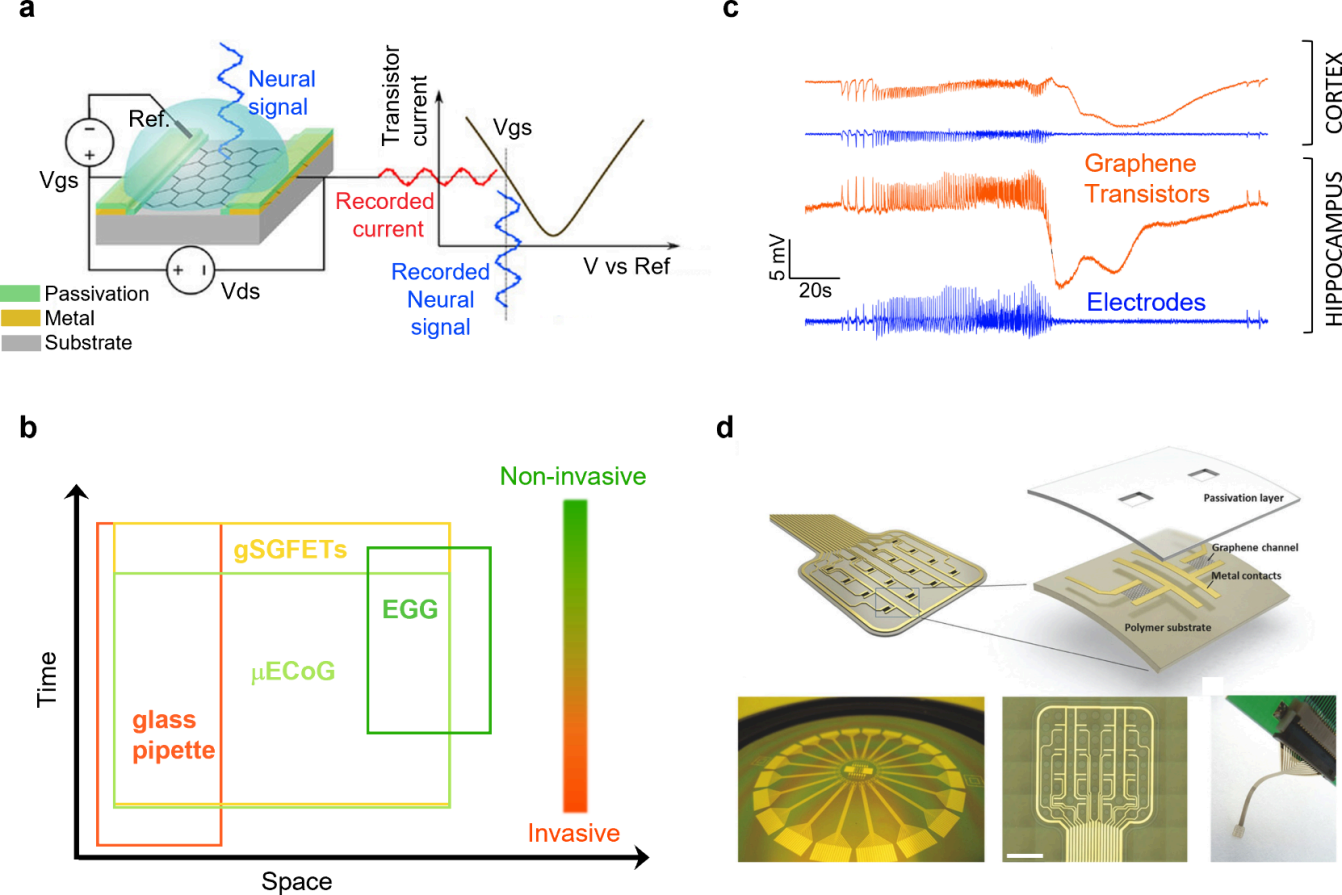
Graphene technology for neural interfacing. (a) Schematic representation of a g-SGFET, showing the driving voltage (Vds) and the operation point selector voltage (Vgs). Current fluctuations in the drain-source current (Ids) are then converted back to voltage using the transfer characteristics of the transistor. Reproduced with permission from ref 5. Copyright 2022 Clinical and Translational Medicine. (b) Conceptual representation of the spatiotemporal regions covered by the different electrophysiological methods. Reproduced with permission from ref 9. Copyright 2019 IEEE. (c) Comparison of in-depth (from cortex to hippocampus) direct current brain potentials recorded with gSGFETs (orange) and electrodes (blue). (d) Schematic of a gSGFET array, showing the different material components, and photographs of a 4 in. wafer containing 20 neural probes (left), a 4 × 4 transistor array (middle), and neural probe used for in vivo validation, connected to a zero-insertion force connector (right). Scale bar is 600 μm. Reproduced with permission from ref 4. Copyright 2017 Advanced Functional Materials.

A unique capability of gSGFETs lies in their ability to capture brain dynamics across the full spectrum of electrophysiological frequencies. Brain activity spans multiple temporal and spatial scales, requiring a set of technologies for its monitoring and to advance its understanding. Several electrophysiological methodologies have been consistently used, providing different spatial resolution with different degrees of invasiveness; see Figure 1b.^9^ Our work has shown that gSGFETs are capable of high-resolution, large-area brain recordings in the whole frequency range of brain activity.^10^ Thanks to the electrochemical stability of graphene and the DC-coupled nature of the transistor-based recordings, gSGFETs can reliably record very slow potential shifts alongside faster oscillatory activity. We have demonstrated these capabilities in diverse neural interfacing configurations from electrocorticography grids to penetrating probes.^11^ As an example, in Figure 1c, we show DC-coupled measurements made with gSGFETs in an epileptic mouse model, revealing the interplay between seizure events and low frequency dynamics across different cortical layers and highlighting the potential of this technology to overcome the limitations of conventional passive electrodes to operate below 0.1 Hz.

The large-scale fabrication of flexible gSGFET probes relies on photolithographic microfabrication adapted from established semiconductor technologies but tailored for application in neurophysiology.^4^ Typical device stacks combine metals, polymers, and graphene layers to form robust yet flexible probes, as illustrated in Figure 1d, which shows a schematic representation of a flexible probe with a 4 × 4 gSGFET array. A central challenge in this process is to preserve graphene quality and properties in the finalized device given its monolayer nature and the incompatibility of harsh cleaning procedures with device integrity. Contaminants introduced during fabrication, such as polymeric residues, remain a persistent issue that degrades the device performance. In this Account, we discuss the implications of such contamination for the operation of gSGFETs and review strategies pursued to mitigate it, ranging from optimized cleaning protocols to protection strategies for graphene. Looking ahead, overcoming these fabrication and material challenges will be critical for translating gSGFET technology from laboratory demonstrations to commercial- or clinical-grade neural interfaces.

## 2 gSGFETs Electrical Characterization

The electrical characterization of gSGFETs relies on measuring the transfer curve (Figure 2a), which represents the drain current (Ids) as a function of the gate-source voltage (Vgs) at a fixed drain-source voltage (Vds). From this curve, key figures of merit can be extracted for benchmarking the device performance. First, the charge neutrality point (CNP), defined as the Vgs at which Ids reaches its minimum, corresponds to the Dirac point, i.e., the Fermi energy when graphene is charge neutral. Owing to the strong sensitivity of two-dimensional (2D) graphene to the surrounding environment, the exact position of the CNP is influenced by substrate interactions and ambient conditions and thus commonly serves as an indicator of the doping state. Another key performance parameter is the transconductance (*g*_m_), defined as the derivative of Ids with respect to Vgs, which reflects the sensitivity of the drain-source current to small variations of the gate voltage.

**Figure 2:**
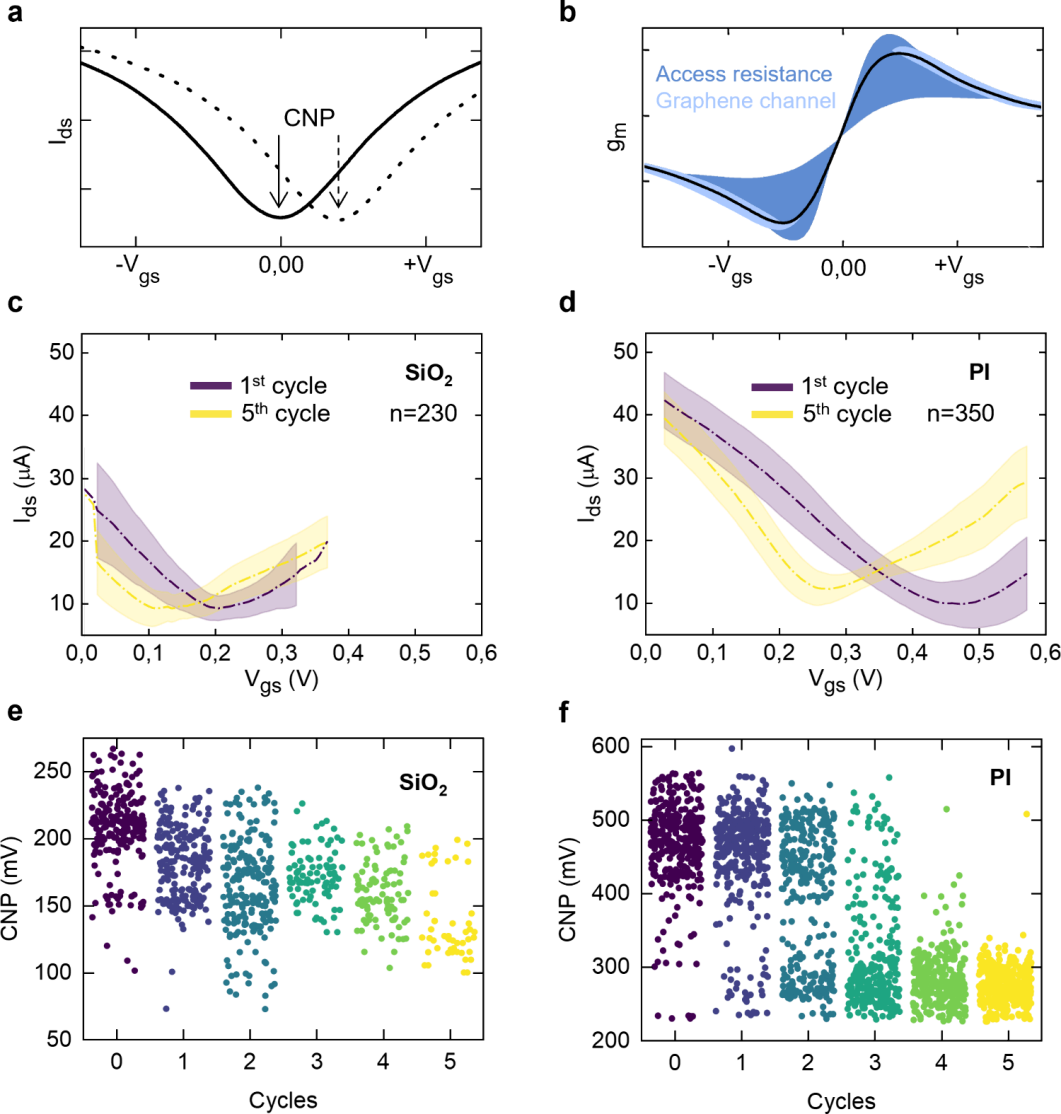
Standard characterization of gSGFETs. (a) Characteristic graphene transfer curve (drain-source current vs gate-source voltage) with the minimum corresponding to the CNP. Dashed line indicates the curve shift induced by n-doping. Adapted from ref 4. (b) Characteristic *g*_m_ vs gate-source voltage, indicating the effect of the access resistance (dark blue) and the graphene channel (light blue). Adapted from ref 4. (c and d) Statistical transfer curves of gSGFETs on Si/SiO_2_ (230 devices) and PI (350 devices), respectively, for the initial (purple) and fifth (yellow) *I*–*V* cycle. (e and f) Statistical CNP drift of gSGFETs on Si/SiO_2_ and PI, respectively. Data derived from refs 10, 11, 28 and 29 .

Whereas the CNP is mainly governed by the intrinsic properties of graphene, *g*_m_ is also determined by the device characteristics like geometry and access resistance.^12,13^ Figure 2b illustrates a representative *g*_m_–Vgs curve of a graphene transistor,^4^ highlighting the influence of access resistance, which includes both the track and the metal–graphene contact resistance. When access resistance is not negligible with respect to the graphene channel resistance, the current response away from the Dirac point flattens, narrowing the linear operating regime of the transistor and reducing its transconductance. Since contact resistance scales with channel width, while channel resistance depends on the width-to-length ratio, optimized device design is crucial to maximize the transconductance. The maximum *g*_m_ value can be used to compare the carrier mobility in graphene devices. To mitigate the effect of the contact resistance, our gSGFET technology incorporates an ultraviolet ozone (UVO) treatment applied at the graphene–metal contact interface.^14^ This strategy effectively reduces contact resistance and improves device linearity, homogeneity, and sensitivity, especially for transistors with large width-to-length ratio.

Due to the complex interfacial phenomena that occur during operation, the benchmarking of gSGFETs remains challenging. Even for devices fabricated with identical processes and characterized under controlled conditions, the transfer characteristics can vary significantly. These variations are dictated by both the intrinsic quality of graphene, such as defects^13^ and residual charges,^15^ and its coupling to the underlying substrate. Substrate-dependent properties, including wettability, permeability, and hydrophilicity, critically influence the graphene–electrolyte interaction and thus the sensing principle of gSGFETs. Some of these interfacial phenomena are dynamic and can lead to a drift in the transfer curve during repeated Vg sweeps (*I*–*V* cycles). This drift has been extensively studied for gSGFETs fabricated on Si/SiO_2_ substrates.^16–18^ Figure 2c shows successive *I*–*V* cycles for over 230 transistors, revealing an initial asymmetry between electron and hole branches (reduced *g*_m_ in the hole branch) and, in some cases, the presence of a double minimum in the transfer curve. Such a double minimum has been suggested to be related to the charge transfer between the graphene and the metal electrode and gets intensified due to the charge storage at the graphene–substrate interface.^19^ With continued Vgs cycling, *g*_m_ increases and the CNP shifts progressively in correspondence with lower doping levels.

While earlier reports attribute the CNP drift primarily to silicon charge trapping,^20,21^ our studies on flexible devices fabricated on polyimide (PI) indicate a more complex behavior. As shown in Figure 2d, gSGFETs on PI (statistical data for more than 350 devices) also experience a variable number of stabilization cycles before reaching steady-state transfer characteristics. This observation suggests that additional interfacial mechanisms beyond charge trapping must be considered: in particular, water^22^ and ion^21^ intercalation between graphene and the substrate (facilitated by graphene grain boundaries), which modifies the graphene–substrate coupling and thus affects the transistor characteristics.^22^ Moreover, redox processes involving trapped water and oxygen molecules can contribute to the evolution of the transfer curve.^21^ Residual charges present an additional degree of complexity: they not only scatter charge carriers in graphene but also affect the electrochemical interaction with the electrolyte. Current annealing, induced by driving current through the graphene channel, can desorb charged species, shifting the CNP toward zero.^23–26^ However, this process does not fully resolve device variability, as different transistors within an array often get stabilized at different CNP values.

As an example, Figure 2e,f illustrates the variability in the CNP across devices fabricated on Si/SiO_2_ and PI substrates, respectively. Dispersion is observed at multiple levels: within a single probe with 48 transistors, across probes fabricated on the same wafer, and between wafers, despite the standardized fabrication protocol (Figure 2e compiles data from two fabrication batches). Even after stabilization (fifth cycle), significant device-to-device variation persists. A survey of the literature (Table S1) further highlights the widespread CNP values reported for gSGFETs fabricated on Si/SiO_2_ substrates under comparable conditions. This lack of homogeneity and reproducibility originates from the inhomogeneous presence of microfabrication-induced residues, which cannot always be completely removed, even after the current annealing process. The persistence of such residues thus represents a key barrier to achieve reliable and scalable graphene neurotechnology. Also, the observed transistor drift has important implications for the specific application of gSGFETs in neurotechnology. Although devices remain operational, a recalibration process is needed by measuring the transfer curve under in vivo conditions and reselecting the optimum bias point for reliable brain recordings. Moreover, in cases of severe contamination, such recalibration could imply biasing out of the graphene potential window, which becomes even narrower if graphene is heavily doped. Also, sensitivity to ions may change if residual charges are present on graphene. Contamination also has a negative impact on graphene’s carrier mobility, reducing the useful range of transconductance and, thus, lowering the efficiency of the voltage-to-current conversion. Contamination can also reduce the effective bandwidth and decrease the gain at high frequency range, impeding the registration of neural spikes. Finally, undesired doping may introduce trap states, increasing the low frequency noise and negatively impacting the sensitivity to low current signals.

## 3 Residual Charge Evaluation and Graphene Cleaning Approaches

Preserving the pristine state of graphene throughout device fabrication remains a major challenge. As a monolayer material, graphene is extremely sensitive to contamination, while standard cleaning protocols are often too harsh and thus are not compatible with this material. In particular, the transfer step has attracted most of the attention. Wet transfer using poly­(methyl methacrylate) (PMMA) remains the most widespread method, and numerous PMMA removal strategies have been reported,^28,29^ including thermal annealing, plasma annealing, photonic and ion-beam techniques, and mechanical cleaning. However, such methods must be applied under mild conditions to avoid damage to graphene, thus limiting their efficacy. Alternative carrier polymers with easier removal, such as paraffin,^30,31^ polydimethylsiloxane (PDMS),^32–34^ poly­(bisphenol A carbonate) (PC),^35,36^ poly­(vinyl alcohol) (PVA),^37–39^ and pentacene,^40^ have been explored. However, due to its favorable viscosity, wetting properties, flexibility, and solubility in common solvents, PMMA continues to be the preferred support layer during transfer, including in industrial processes.^41^ In all cases, however, transfer-related cleaning protocols are difficult to standardize and remain challenging to scale. Most recently, efforts have shifted toward direct growth of graphene on the target substrate, an approach that not only avoids the transfer step but also addresses issues related to substrate availability. Successful demonstrations include growth on SiO_2_,^42–44^ Al_2_O_3_,^45^ and flexible polymers^46^ such as PI and PDMS.

Yet, the graphene transfer is only the first step of the microfabrication process, but graphene is subsequently in contact with multiple resins, polymers, and solvents. Since the presence of residual charges critically impacts gSGFET device performance, methodologies to monitor the doping state of graphene throughout fabrication are crucial. Unfortunately, the gSGFET transfer curve is not accessible until the device is finalized, so alternative methods must be employed during processing. Among the nondestructive common characterization techniques, thanks to the strong electron–phonon coupling in graphene, Raman spectroscopy can be used to estimate the charge state of graphene and therefore to the density of adsorbed residues (σ) on its surface (see the SI for more details).

Uncontrolled and inhomogeneous contamination on the graphene surface introduced by photolithographic microfabrication represents a bottleneck for advancing gSGFETs technology. Effective, scalable, and material-compatible cleaning procedures are therefore required. A literature survey (Figure 3a) shows a variety of graphene cleaning approaches, as the aforementioned current annealing (Figure 3b).^23^ Mechanical cleaning with atomic force microscopy (AFM) tips can also effectively remove residues (Figure 3c)^47^ but is limited by its lack of scalability and throughput for wafer-scale production of gSGFETs. High-temperature annealing (Figure 3d) is also widely adopted, as it effectively removes polymer residues and can restore graphene’s intrinsic properties, often yielding CNP values near 0 V.^48^ Nonetheless, high temperature can induce defects degrading the electronic quality of graphene, particularly under harsh conditions.

**Figure 3:**
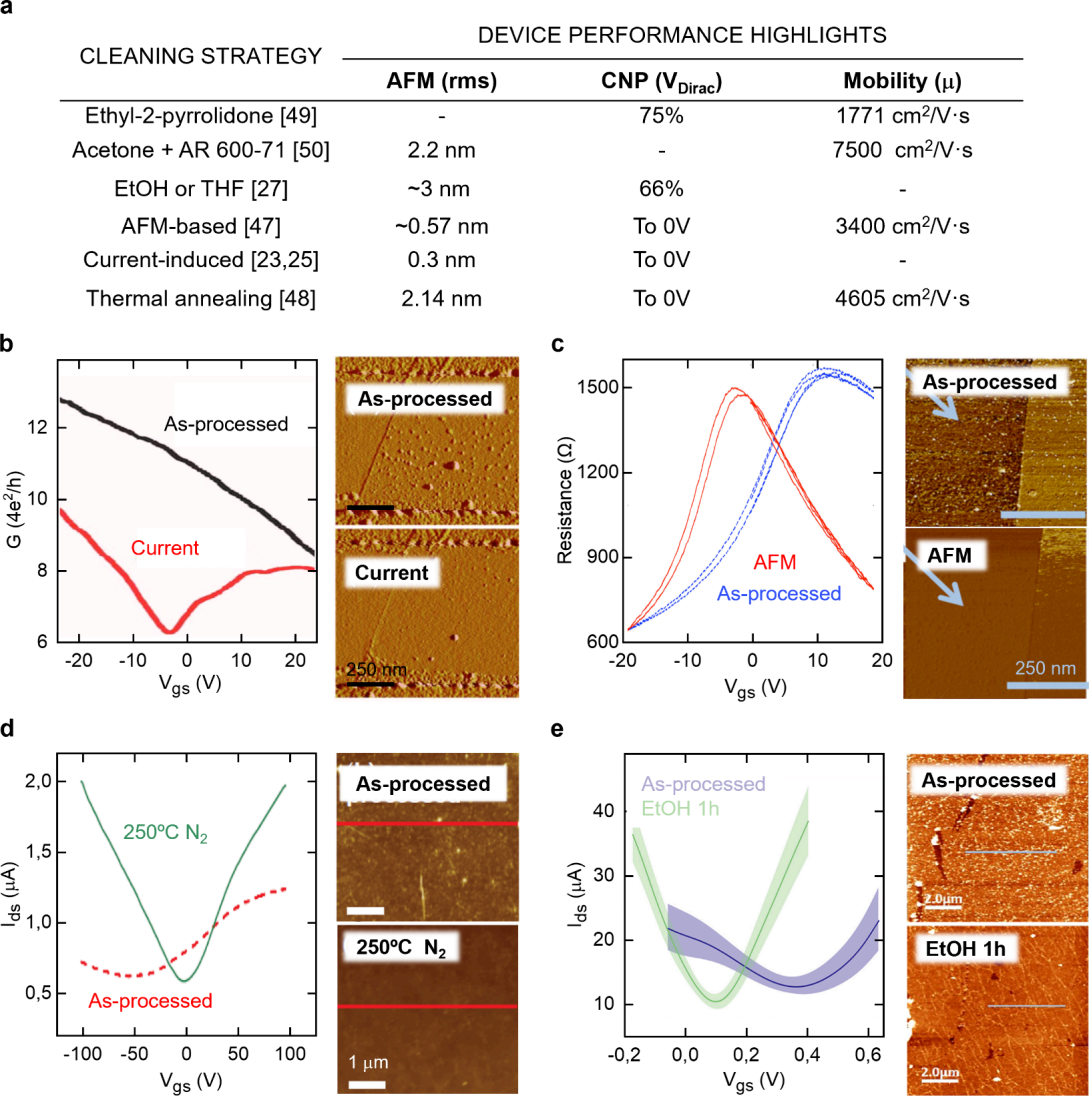
Cleaning strategies. (a) Bibliographic review of device improvement: AFM roughness (rms) shift, CNP reduction percentage, and maximum mobility reported after cleaning. AFM images and electrical characterization of graphene devices before and after cleaning step: (b) Current annealing (scale bar of 250 nm). Adapted with permission from ref 23. Copyright 2007 Applied Physics Letters. (c) Mechanical AFM cleaning (scale bar of 250 nm). Adapted with permission from ref 47. Copyright 2012 Journal of Applied Physics. (d) Thermal annealing (scale bar of 1 μm). Adapted with permission from ref 48. Copyright 2013 Nanotechnology. (e) Chemical cleaning (scale bar of 2 μm). Adapted with permission from ref 27. Copyright 2024 The Authors. Published by American Chemical Society.^49,50^

We have also explored graphene cleaning with organic solvents after fabrication (Figure 3e).^27^ Comparing ethanol (EtOH) and tetrahydrofuran (THF), we concluded that both solvents are effective at reducing residues. However, THF compromises the structural integrity of graphene and the passivation layer in gSGFETs. In contrast, EtOH is fully compatible with all device materials and is scalable to wafer-level processing. EtOH cleaning induces a clear undoping effect, shifting the transfer curve minimum toward zero and improving device homogeneity. Nevertheless, the efficiency of this method is limited by the intrinsic inhomogeneity of residue deposition, which varies even among devices from the same fabrication batch. As shown by Raman analysis (Figure S1), σ variability persists after EtOH treatment, highlighting that solvent cleaning alone cannot fully ensure reproducibility. Developing strategies to mitigate contamination during photolithography will be essential for improving the device yield and performance.

## 4 Graphene Protection Strategies

One of the most promising strategies for preserving graphene during device fabrication is the deposition of a sacrificial protective layer. This additional layer isolates graphene from direct contact with polymers, resins, and solvents throughout the fabrication process and is removed after the final step. Among the various candidates, thin metal layers deposited by e-beam evaporation have been extensively studied. Under optimized deposition conditions, detrimental effects such as bond breaking or metal penetration into the 2D material can be minimized, thereby avoiding structural degradation of graphene.^51^ In fact, evaporating metals under moderate vacuum has been found to preserve the integrity of the 2D material, leading to nearly perfect van der Waals metal contacts. For example, cross-sectional transmission electron microscopy (TEM) and energy dispersive X-ray spectroscopy (EDS) analysis of gold (Au) deposited on hexagonal boron nitride (hBN) at 5 × 10^–6^ Torr reveal no metal infiltration or atomic distortion, in contrast to samples evaporated at higher pressure. The authors proposed that at pressures above 10^–6^ Torr residual water molecules on the 2D surface can distort the lattice, weaken the covalent bonds, and lower the energy barrier for defect formation.

The use of Au as a sacrificial protective layer was the first to be reported, showing improvement in device performance, such as enhanced mobility, reduced defect density, and lower unintentional doping, contact resistance, and sheet resistance.^52^ Titanium (Ti) was later studied as an alternative sacrificial layer^53^ to improve the graphene technology in terms of both yield and sheet resistance. More recently, aluminum (Al) has been implemented as an intermediate sacrificial layer, yielding enhanced consistency, mobility, and conductivity in graphene devices.^54,55^ The advantages of these metal layers lie in their technological maturity and relatively low cost. However, their complete removal after fabrication without damaging graphene or other materials composing the electronic device still remains challenging. For instance, Au requires aggressive oxidative solutions that may damage graphene, while Ti removal relies on hydrofluoric acid (HF), making it incompatible with silicon oxide substrates. In the case of Al, partial oxidation is often unavoidable, and recent strategies focus on avoiding the final metal etching and depositing a final 30 nm alumina (Al_2_O_3_) passivation layer.^56^ This approach leads to a clear improved control of the graphene conductance, also helping the advance of this technology in terms of homogeneity, yield, and reproducibility. However, for neural applications, this approach would impede the direct graphene contact with the tissue, thus diminishing the device sensitivity.

Yttrium (Y) has also been explored as a sacrificial protective layer.^57^ Devices processed with Y show markedly improved electrical characteristics, including transfer curves with minima close to zero, enhanced *g*_m_, significant reduction in contact resistance (2.5× lower), and mobility improvement (3× higher), when compared to nonprotected devices. Importantly, Y layers can be efficiently removed using diluted hydrochloric acid (HCl) without observable damage to graphene, contacts, or substrate. Nevertheless, the use of Y is limited by its scarcity, cost, and environmental as well as health hazards, making it unsuitable for scalable and standardized high-throughput device fabrication.

Here, we propose the use of copper (Cu) as a sacrificial protective layer. The main advantage of Cu is that its etching chemistry is already well established in graphene transfer processes as Cu is the catalytic substrate most commonly used for graphene growth. Several mild solutions have been demonstrated to effectively remove Cu, while preserving graphene properties. Residual Cu concentrations after etching are relatively low (10^13^ atoms per cm^2^) and do not significantly affect the back-end-of-line integration of graphene devices.^58^

Adapting this approach to our standardized fabrication process implied refinement of established protocols, since previous studies^53–55^ noted that metal protective layers can lengthen the resist development times, potentially leading to overdevelopment and yield loss in fine features. To assess the viability of Cu as a sacrificial layer, we fabricated graphene macrotransistors, following the protocol depicted in Figure 4a, which enable the evaluation of each photolithography step, including as-transfer graphene devices. After graphene transfer, a 20 nm-thick Cu layer was evaporated under ultrahigh vacuum (∼10^–7^ mbar) to prevent graphene damage.^51^ AFM and scanning electron microscopy (SEM) confirmed the formation of a continuous Cu film covering the graphene surface (see Figure S2 for more details). Following fabrication, the Cu layer was etched using ammonium persulfate, APS ((NH_4_)_2_S_2_O_8_), at 0.02 g/mL. X-ray photoelectron spectroscopy (XPS) and cyclic voltammetry (CV) revealed the complete removal of Cu after 7 min of etching, as further confirmed by Time-of-Flight Secondary Ion Mass Spectrometry (ToF-SIMS); see Figure S3 for further details.

**Figure 4:**
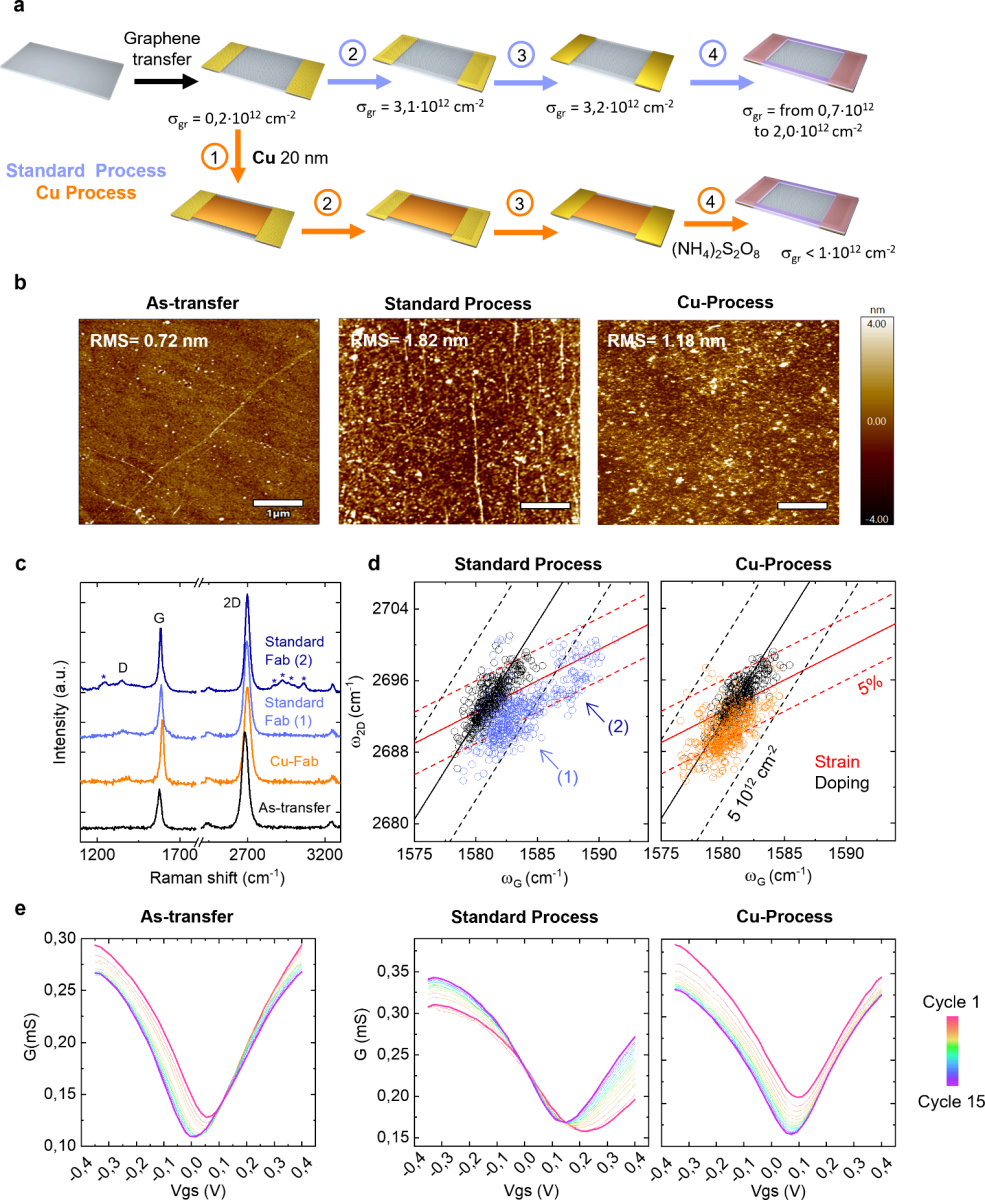
Graphene protection strategy with a Cu sacrificial layer. Characterization of gSGFETS after transfer, standard and Cu-protected processes. (a) Schematic representation of gSGFETs microfabrication, including the standard process (blue) and the Cu protection process (orange). Estimated σ_gr_ (according to Figure S1) for each fabrication stage. Fabrication steps: (1) Cu 20 nm deposition, (2) graphene definition, (3) top metal definition, and (4) device passivation and EtOH cleaning. An additional Cu etching step (7 min (NH_4_)_2_S_2_O_8_) is added for the Cu process. (b) AFM topography images (scale bar of 1 μm). (c) Representative Raman spectra. (d) Raman correlation maps (ω_2D_ vs ω_G_). (e) Transistor transfer curve (G vs Vgs for Vds = 50 mV) evolution.

The beneficial effect of Cu protection on graphene quality is clearly evidenced in the AFM topography (Figure 4b). Graphene processed with the standard protocol exhibits significant residue accumulation and a higher roughness compared to as-transfer graphene, while Cu-protected devices show reduced contamination and smoother surfaces. Nevertheless, some residues remain in Cu-protected devices, likely originated from polymers adhering to the Cu surface and redepositing during the Cu etching step. This highlights the importance of combining sacrificial protection with optimized additional cleaning steps during fabrication to eliminate residual adsorbates. Notably, the use of sacrificial protective layers enables the use of harsher cleaning protocols that would otherwise damage directly exposed graphene.

Raman spectroscopy further confirms the advantages of Cu protection (Figure 4c,d). Correlation maps of the 2D and the G band Raman shift, typically used to discern between strain and doping in graphene,^59^ reveal that, while as-transfer graphene shows the expected native strain gradient across the sampling area, standard fabrication induces substantial doping level (∼3 × 10^12^ cm^–2^) and nonhomogeneous residue accumulation, consistent with characteristic polymer Raman features appreciated in the corresponding spectrum (Figure 4c dark and light blue spectra). In contrast, Cu-protected devices exhibit only strain-related modifications, likely due to partial decoupling of graphene from the substrate after Cu deposition, as suggested by the 2D band downshift. Importantly, in contrast to devices fabricated following the standard protocol, which present great dispersion in terms of charged residues, with σ ranging from ∼0.7 × 10^12^ to 2.1 × 10^12^ cm^–2^ (estimated according to Figure S1), in Cu-protected graphene, residual charge doping is homogeneous and remains below 1 × 10^12^ cm^–2^, a marked improvement.

Electrical measurements corroborate these findings (Figure 4e). As-transfer graphene devices exhibit symmetric transfer curves centered near ∼50 mV with minimal drift upon cycling. Eventually, the CNPs are stabilized at ∼10 mV, slightly shifted from zero, and mostly associated with coupling with the substrate. Devices fabricated with the standard protocol display an initial asymmetric transfer curve, reduced *g*_m_, and broadened minimum, shifted to ∼200 mV. These features (including in cases of severe contamination a characteristic double dip in the hole branch, not observed for as-transfer graphene nor for Cu processes devices, despite having identical substrate and contact structure), clearly evidence the presence of negatively charged residues that scatter positive carriers, limiting their mobility.^60,61^ Vgs cycling partially mitigates these effects through current annealing (more symmetric transfer curve stabilized at ∼150 mV), but reproducibility issues persist (Figure S4). In Cu-protected devices, although a small residual doping remains (already observed by means of AFM and Raman spectroscopy and expected to mitigate when implementing the aforementioned additional cleaning step), Vgs cycling shifts the CNP position from ∼120 mV to ∼90 mV, improving the working potential window. More importantly, the Cu protection strategy leads to devices exhibiting initial symmetric transfer curves, transconductance comparable to as-transfer graphene, and uniform behavior across multiple devices, confirming the robustness of the Cu protection approach. Device reproducibility (CNP statistics) is presented in Figure S4.

## 5 Conclusions and Perspectives

Graphene presents a unique combination of properties that position it as a material of great potential for neurotechnology. Recent improvement in the maturity level of graphene-based thin film technology has demonstrated its ability to achieve high-fidelity, wide-bandwidth brain recordings, validating its potential for next-generation neural interfaces. However, the upscaling and standardization of graphene technology necessarily rely on photolithography-based microfabrication, which often introduces defects and residual charges. Given the 2D nature of graphene, restoring the pristine material quality after microfabrication represents a huge challenge.

Unwanted chemical dopants not only alter the electronic response of graphene and contribute to the variability in device performance frequently reported in the literature but also can impair biocompatibility as well as long-term stability and reliability. Process-engineering efforts are being directed toward mitigating this issue, including (i) transfer-free graphene synthesis or cleaner transfer methods, which nevertheless do not prevent contamination introduced during subsequent fabrication steps, and (ii) interfacial encapsulation or surface modification, which still require a pristine graphene surface to achieve high performance. Postfabrication cleaning protocols have also been proposed, yet reproducibility and consistency in graphene technologies remain challenging. Novel solutions are therefore needed together with appropriate characterization benchmarks and comparability metrics. Among emerging approaches, sacrificial protective layers have been proven to be especially promising as effective barriers against process-induced contamination. Our work highlights the potential of Cu as a sacrificial protective layer, owing to its compatibility with established graphene transfer strategies and the availability of mild etchants for its removal.

Further efforts are needed to integrate the sacrificial protection strategies in the standardized microfabrication workflows to advance graphene technology toward high-yield and large-scale production. Achieving this level of process reliability will not only accelerate the translation of graphene-based neural interfaces but also strengthen the graphene position as a key material platform for a broad range of bioelectronics and nanoelectronic applications.

## Supplementary Material



## Notes

Supplementary Note 1: Materials and methods; experimental details for device morphological (Raman spectroscopy and AFM) and electrical characterization; Supplementary Note 2: Additional bibliographic review; reported CNP values for gSGFETs; Supplementary Note 3: Graphene charge carriers estimation by Raman spectroscopy; charge equilibrium at graphene interfaces; principle and Raman study in ionic solution; strength variation; Supplementary Note 4: Protection strategies review; device performance highlights for protective strategies; Supplementary Note 5: Cu protection additional characterization; Cu layer surface characterization by AFM and SEM; Cu etching evidence by XPS, CV and TOF-SIMS; Supplementary Note 6: Electrical characterization; comparison and CNP statistics for standard and Cu-protected fabricated macrotransistors (PDF)

## References

[1] Hess L. H., Jansen M., Maybeck V., Hauf M. V., Seifert M., Stutzmann M., Sharp I. D., Offenhäusser A., Garrido J. A. (2011). Graphene Transistor Arrays for Recording Action Potentials
from Electrogenic Cells. Adv. Mater..

[2] Blaschke B. M., Tort-Colet N., Guimerà-Brunet A., Weinert J., Rousseau L., Heimann A., Drieschner S., Kempski O., Villa R., Sanchez-Vives M. V., Garrido J. A. (2017). Mapping Brain Activity with Flexible Graphene Micro-Transistors. 2D Mater..

[3] Hess L. H., Seifert M., Garrido J. A. (2013). Graphene Transistors
for Bioelectronics. Proc. IEEE.

[4] Hébert C., Masvidal-Codina E., Suarez-Perez A., Bonaccini Calia A., Piret G., Garcia-Cortadella R., Illa X., Del Corro
Garcia E., De la Cruz Sanchez J.
M., Viana Casals D., Prats-Alfonso E., Bousquet J., Godignon P., Yvert B., Villa R., Sanchez-Vives M. V., Guimerà-Brunet A., Garrido J. A. (2018). Flexible Graphene Solution-Gated Field-Effect Transistors
Efficient Transducers for microelectrocorticography. Adv. Funct. Mater..

[5] Wykes R. C, Masvidal-Codina E., Guimera-Brunet A., Garrido J. A (2022). The Advantages of
Mapping Slow Brain Potentials Using DC-coupled Graphene Micro-transistors:
Clinical and Translational Applications. Clin.
Transl. Med..

[6] Garcia-Cortadella R., Schäfer N., Cisneros-Fernandez J., Ré L., Illa X., Schwesig G., Moya A., Santiago S., Guirado G., Villa R., Sirota A., Serra-Graells F., Garrido J. A., Guimerà-Brunet A. (2020). Switchless Multiplexing
of Graphene Active Sensor Arrays for Brain Mapping. Nano Lett..

[7] Cisneros-Fernandez J., Garcia-Cortadella R., Illa X., Martinez-Aguilar J., Paetzold J., Mohrlok R., Kurnoth M., Jeschke C., Teres L., Garrido J. A., Guimera-Brunet A., Serra-Graells F. (2021). A 1024-Channel 10-Bit 36- μW/Ch
CMOS ROIC for
Multiplexed GFET-Only Sensor Arrays in Brain Mapping. IEEE Trans Biomed Circuits Syst..

[8] Garcia-Cortadella R., Schwesig G., Jeschke C., Illa X., Gray A. L., Savage S., Stamatidou E., Schiessl I., Masvidal-Codina E., Kostarelos K., Guimerà-Brunet A., Sirota A., Garrido J. A. (2021). Graphene
Active Sensor Arrays for Long-Term and Wireless
Mapping of Wide Frequency Band Epicortical Brain Activity. Nat. Commun..

[9] Guimera-Brunet, A. ; Masvidal-Codina, E. ; Illa, X. ; Dasilva, M. ; Bonaccini-Calia, A. ; Prats-Alfonso, E. ; Martinez-Aguilar, J. ; De La Cruz, J. M. ; Garcia-Cortadella, R. ; Schaefer, N. ; Barbero, A. ; Godignon, P. ; Rius, G. ; Del Corro, E. ; Bousquet, J. ; Hebert, C. ; Wykes, R. ; Sanchez-Vives, M. V. ; Villa, R. ; Garrido, J. A. Neural Interfaces Based on Flexible Graphene Transistors: A New Tool for Electrophysiology. In 2019 IEEE International Electron Devices Meeting (IEDM); IEEE: San Francisco, CA, USA, 2019; p 18.3.1–18.3.4; 10.1109/IEDM19573.2019.8993433.

[10] Masvidal-Codina E., Illa X., Dasilva M., Calia A. B., Dragojević T., Vidal-Rosas E. E., Prats-Alfonso E., Martínez-Aguilar J., De La Cruz J. M., Garcia-Cortadella R., Godignon P., Rius G., Camassa A., Del Corro E., Bousquet J., Hébert C., Durduran T., Villa R., Sanchez-Vives M. V., Garrido J. A., Guimerà-Brunet A. (2019). High-Resolution Mapping
of Infraslow Cortical Brain Activity Enabled by Graphene Microtransistors. Nat. Mater..

[11] Bonaccini
Calia A., Masvidal-Codina E., Smith T. M., Schäfer N., Rathore D., Rodríguez-Lucas E., Illa X., De La Cruz J. M., Del Corro E., Prats-Alfonso E., Viana D., Bousquet J., Hébert C., Martínez-Aguilar J., Sperling J. R., Drummond M., Halder A., Dodd A., Barr K., Savage S., Fornell J., Sort J., Guger C., Villa R., Kostarelos K., Wykes R. C., Guimerà-Brunet A., Garrido J. A. (2022). Full-Bandwidth Electrophysiology of Seizures and Epileptiform
Activity Enabled by Flexible Graphene Microtransistor Depth Neural
Probes. Nat. Nanotechnol..

[12] Hwang B.-W., Yeom H.-I., Kim D., Kim C.-K., Lee D., Choi Y.-K. (2018). Enhanced Transconductance
in a Double-Gate Graphene
Field-Effect Transistor. Solid-State Electron..

[13] Jiménez D., Cummings A. W., Chaves F., Van Tuan D., Kotakoski J., Roche S. (2014). Impact of Graphene
Polycrystallinity on the Performance of Graphene
Field-Effect Transistors. Appl. Phys. Lett..

[14] Schaefer N., Garcia-Cortadella R., Calia A. B., Mavredakis N., Illa X., Masvidal-Codina E., Cruz J. D. L., Corro E. D., Rodríguez L., Prats-Alfonso E., Bousquet J., Martínez-Aguilar J., Pérez-Marín A. P., Hébert C., Villa R., Jiménez D., Guimerà-Brunet A., Garrido J. A. (2020). Improved Metal-Graphene Contacts for Low-Noise, High-Density
Microtransistor Arrays for Neural Sensing. Carbon.

[15] Mercado E., Anaya J., Kuball M. (2021). Impact of
Polymer Residue Level on
the In-Plane Thermal Conductivity of Suspended Large-Area Graphene
Sheets. ACS Appl. Mater. Interfaces.

[16] Kireev D., Brambach M., Seyock S., Maybeck V., Fu W., Wolfrum B., Offenhäusser A. (2017). Graphene Transistors for Interfacing
with Cells: Towards a Deeper Understanding of Liquid Gating and Sensitivity. Sci. Rep..

[17] Miyakawa N., Shinagawa A., Kajiwara Y., Ushiba S., Ono T., Kanai Y., Tani S., Kimura M., Matsumoto K. (2021). Drift Suppression
of Solution-Gated Graphene Field-Effect Transistors by Cation Doping
for Sensing Platforms. Sensors.

[18] Mouro J., Domingues T., Pereira T., Campos R., Borme J., Alpuim P. (2025). Analytical
Modeling and Experimental Characterization
of Drift in Electrolyte-Gated Graphene Field-Effect Transistors. Npj 2D Mater. Appl..

[19] Bartolomeo A. D., Giubileo F., Santandrea S., Romeo F., Citro R., Schroeder T., Lupina G. (2011). Charge Transfer and Partial Pinning
at the Contacts as the Origin of a Double Dip in the Transfer Characteristics
of Graphene-Based Field-Effect Transistors. Nanotechnology.

[20] Lee Y. G., Kang C. G., Jung U. J., Kim J. J., Hwang H. J., Chung H.-J., Seo S., Choi R., Lee B. H. (2011). Fast Transient
Charging at the Graphene/SiO2 Interface Causing Hysteretic Device
Characteristics. Appl. Phys. Lett..

[21] Feng T., Xie D., Li G., Xu J., Zhao H., Ren T., Zhu H. (2014). Temperature and Gate
Voltage Dependent Electrical Properties of Graphene
Field-Effect Transistors. Carbon.

[22] Delgà-Fernández M., Toral-Lopez A., Guimerà-Brunet A., Pérez-Marín A. P., Marin E. G., Godoy A., Garrido J. A., Del Corro E. (2024). Interfacial
Phenomena Governing Performance of Graphene Electrodes in Aqueous
Electrolyte. Nano Lett..

[23] Moser J., Barreiro A., Bachtold A. (2007). Current-Induced
Cleaning of Graphene. Appl. Phys. Lett..

[24] Prasad N., Kumari A., Bhatnagar P. K., Mathur P. C., Bhatia C. S. (2014). Current
Induced Annealing and Electrical Characterization of Single Layer
Graphene Grown by Chemical Vapor Deposition for Future Interconnects
in VLSI Circuits. Appl. Phys. Lett..

[25] Ramamoorthy H., Somphonsane R. (2018). In-Situ Current
Annealing of Graphene-Metal Contacts. J. Phys.
Conf. Ser..

[26] Brosel-Oliu S., Rius G., Avino A., Nakatsuka N., Illa X., del Corro E., Delga-Fernandez M., Masvidal-Codina E., Rodriguez N., Merino J. P., Criado A., Prato M., Tkatchenko R., Eritja R., Godignon P., Garrido J. A., Villa R., Guimera A., Prats-Alfonso E. (2024). Single-Step
Functionalization Strategy of Graphene Microtransistor Array with
Chemically Modified Aptamers for Biosensing Applications. Small.

[27] Merino J. P., Brosel-Oliu S., Rius G., Illa X., Sulleiro M. V., Del Corro E., Masvidal-Codina E., Bonaccini Calia A., Garrido J. A., Villa R., Guimerà-Brunet A., Prato M., Criado A., Prats-Alfonso E. (2024). Ethanol Solvation
of Polymer Residues in Graphene Solution-Gated Field Effect Transistors. ACS Sustain. Chem. Eng..

[28] Zhuang B., Li S., Li S., Yin J. (2021). Ways to Eliminate PMMA Residues on
Graphene - Superclean Graphene. Carbon.

[29] Dong W., Dai Z., Liu L., Zhang Z. (2024). Toward Clean 2D Materials and Devices:
Recent Progress in Transfer and Cleaning Methods. Adv. Mater..

[30] Leong W. S., Wang H., Yeo J., Martin-Martinez F. J., Zubair A., Shen P.-C., Mao Y., Palacios T., Buehler M. J., Hong J.-Y., Kong J. (2019). Paraffin-Enabled Graphene
Transfer. Nat. Commun..

[31] Nam J.-U., Kim B.-H., Hong S.-J., Jeon G.-H., Park J.-W., Kim U. J., Choi Y.-S., Woo Y. S. (2025). Toward Selecting
Optimal Support Layer for CVD-Grown Graphene Transfer onto Arbitrary
Substrate by Surface Energy Engineering. Appl.
Surf. Sci..

[32] Jeong S., Jung M.-W., Lee J.-Y., Kim H., Lim J., An K.-S., Choi Y., Lee S. S. (2013). Graphene
Electrodes
Transfer-Printed with a Surface Energy-Mediated Wet PDMS Stamp: Impact
of Au Doped-Graphene for High Performance Soluble Oxide Thin-Film
Transistors. J. Mater. Chem. C.

[33] Vaziri, S. ; Smith, A. D. ; Lupina, G. ; Lemme, M. C. ; Ostling, M. PDMS-Supported Graphene Transfer Using Intermediary Polymer Layers. In 2014 44th European Solid State Device Research Conference (ESSDERC); IEEE: Venice Lido, Italy, 2014; pp 309–312; 10.1109/ESSDERC.2014.6948822.

[34] Lee J.-H., Lee H.-B., Jeong N. B., Park D.-H., Choi I., Chung H.-J. (2020). High-Speed Residue-Free Transfer of Two-Dimensional
Materials Using PDMS Stamp and Water Infiltration. Curr. Appl. Phys..

[35] Wood J. D., Doidge G. P., Carrion E. A., Koepke J. C., Kaitz J. A., Datye I., Behnam A., Hewaparakrama J., Aruin B., Chen Y., Dong H., Haasch R. T., Lyding J. W., Pop E. (2015). Annealing Free, Clean
Graphene Transfer
Using Alternative Polymer Scaffolds. Nanotechnology.

[36] Lin Y.-C., Jin C., Lee J.-C., Jen S.-F., Suenaga K., Chiu P.-W. (2011). Clean Transfer
of Graphene for Isolation and Suspension. ACS
Nano.

[37] Van
Ngoc H., Qian Y., Han S. K., Kang D. J. (2016). PMMA-Etching-Free
Transfer of Wafer-Scale Chemical Vapor Deposition Two-Dimensional
Atomic Crystal by a Water Soluble Polyvinyl Alcohol Polymer Method. Sci. Rep..

[38] Shivayogimath A., Whelan P. R., Mackenzie D. M. A., Luo B., Huang D., Luo D., Wang M., Gammelgaard L., Shi H., Ruoff R. S., Bøggild P., Booth T. J. (2019). Do-It-Yourself Transfer of Large-Area
Graphene Using an Office Laminator and Water. Chem. Mater..

[39] Li Y., Weng S., Niu R., Zhen W., Xu F., Zhu W., Zhang C. (2022). Poly­(Vinyl Alcohol)-Assisted Exfoliation of van Der
Waals Materials. ACS Omega.

[40] Kim H. H., Kang B., Suk J. W., Li N., Kim K. S., Ruoff R. S., Lee W. H., Cho K. (2015). Clean Transfer
of Wafer-Scale
Graphene *via* Liquid Phase Removal of Polycyclic Aromatic
Hydrocarbons. ACS Nano.

[41] Jiao L., Fan B., Xian X., Wu Z., Zhang J., Liu Z. (2008). Creation of
Nanostructures with Poly­(Methyl Methacrylate)-Mediated Nanotransfer
Printing. J. Am. Chem. Soc..

[42] Ahn Y., Kim J., Ganorkar S., Kim Y.-H., Kim S.-I. (2016). Thermal Annealing
of Graphene to Remove Polymer Residues. Mater.
Express.

[43] Bi H., Sun S., Huang F., Xie X., Jiang M. (2012). Direct Growth of Few-Layer
Graphene Films on SiO_2_ Substrates and Their Photovoltaic
Applications. J. Mater. Chem..

[44] Xu S. C., Man B. Y., Jiang S. Z., Chen C. S., Yang C., Liu M., Gao X. G., Sun Z. C., Zhang C. (2013). Direct Synthesis of
Graphene on SiO2 Substrates by Chemical Vapor Deposition. CrystEngComm.

[45] Hwang J., Kim M., Campbell D., Alsalman H. A., Kwak J. Y., Shivaraman S., Woll A. R., Singh A. K., Hennig R. G., Gorantla S., Rümmeli M. H., Spencer M. G. (2013). Van Der Waals Epitaxial Growth of
Graphene on Sapphire by Chemical Vapor Deposition without a Metal
Catalyst. ACS Nano.

[46] Pham, V. P. Direct Growth of Graphene on Flexible Substrates toward Flexible Electronics: A Promising Perspective. In Flexible Electronics; Rackauskas, S. , Ed.; InTech, 2018; 10.5772/intechopen.73171.

[47] Lindvall N., Kalabukhov A., Yurgens A. (2012). Cleaning Graphene Using Atomic Force
Microscope. J. Appl. Phys..

[48] Jang C. W., Kim J. H., Kim J. M., Shin D. H., Kim S., Choi S.-H. (2013). Rapid-Thermal-Annealing
Surface Treatment for Restoring
the Intrinsic Properties of Graphene Field-Effect Transistors. Nanotechnology.

[49] Shi R., Xu H., Chen B., Zhang Z., Peng L.-M. (2013). Scalable Fabrication
of Graphene Devices through Photolithography. Appl. Phys. Lett..

[50] Tyagi A., Mišeikis V., Martini L., Forti S., Mishra N., Gebeyehu Z. M., Giambra M. A., Zribi J., Frégnaux M., Aureau D., Romagnoli M., Beltram F., Coletti C. (2022). Ultra-Clean
High-Mobility Graphene on Technologically Relevant Substrates. Nanoscale.

[51] Zheng W., Yuan B., Villena M. A., Zhu K., Pazos S., Shen Y., Yuan Y., Ping Y., Liu C., Zhang X., Zhang X., Lanza M. (2024). The Origin and Mitigation
of Defects Induced by Metal Evaporation in 2D Materials. Mater. Sci. Eng. R Rep..

[52] Choi J., Kim H., Park J., Iqbal M. W., Iqbal M. Z., Eom J., Jung J. (2014). Enhanced Performance of Graphene by Using Gold Film for Transfer
and Masking Process. Curr. Appl. Phys..

[53] Theofanopoulos P. C., Ageno S., Guo Y., Kale S., Wang Q. H., Trichopoulos G. C. (2019). High-Yield Fabrication Method for High-Frequency Graphene
Devices Using Titanium Sacrificial Layers. J.
Vac. Sci. Technol. B Nanotechnol. Microelectron. Mater. Process. Meas.
Phenom..

[54] Wang J., Wang Y., Su N., Li M. (2023). Improving Consistency
and Performance of Graphene-Based Devices via Al Sacrificial Layer. Colloid Interface Sci. Commun..

[55] Wang Y., Su N., Wei S., Wang J., Li M. (2024). Enhancing the Consistency
and Performance of Graphene-Based Devices via Al Intermediate-Layer-Assisted
Transfer and Patterning. Nanomaterials.

[56] Mzali S., Montanaro A., Xavier S., Servet B., Mazellier J.-P., Bezencenet O., Legagneux P., Piquemal-Banci M., Galceran R., Dlubak B., Seneor P., Martin M.-B., Hofmann S., Robertson J., Cojocaru C.-S., Centeno A., Zurutuza A. (2016). Stabilizing a Graphene Platform toward Discrete Components. Appl. Phys. Lett..

[57] Wang N. C., Carrion E. A., Tung M. C., Pop E. (2017). Reducing Graphene Device
Variability with Yttrium Sacrificial Layers. Appl. Phys. Lett..

[58] Lupina G., Kitzmann J., Costina I., Lukosius M., Wenger C., Wolff A., Vaziri S., Östling M., Pasternak I., Krajewska A., Strupinski W., Kataria S., Gahoi A., Lemme M. C., Ruhl G., Zoth G., Luxenhofer O., Mehr W. (2015). Residual Metallic Contamination
of Transferred Chemical Vapor Deposited Graphene. ACS Nano.

[59] Lee J. E., Ahn G., Shim J., Lee Y. S., Ryu S. (2012). Optical Separation
of Mechanical Strain from Charge Doping in Graphene. Nat. Commun..

[60] Novikov D. S. (2007). Numbers
of Donors and Acceptors from Transport Measurements in Graphene. Appl. Phys. Lett..

[61] Hwang E. H., Adam S., Sarma S. D. (2007). Carrier Transport in Two-Dimensional
Graphene Layers. Phys. Rev. Lett..

