# Supplementary material for: Mitigating Interfacial Contamination for Scalable Integration of Graphene in Neuroelectronic Devices
Source: Acc Mater Res. 2026 Feb 20;7(4):330–40. doi: 10.1021/accountsmr.5c00259 (PMC13131312; doi:10.1021/accountsmr.5c00259)
Supplement: Supplementary file 1 [file mr5c00259_si_001.pdf]

Supporting information

Mitigating Interfacial Contamination for Scalable Integration of Graphene in Neuroelectronic Devices

*Aina Galceran[1], Marta Delgà-Fernández[1], Xavier Illa[2,3], Anton Guimerà-Brunet[2,3], Jose A. Garrido[1,4] and Elena del Corro[1]**

[1]Catalan Institute of Nanoscience and Nanotechnology (ICN2), CSIC and BIST, 08193 Bellaterra, Spain
[2]Institut de Microelectrònica de Barcelona (IMB-CNM), CSIC, Esfera UAB, 08193 Bellaterra, Spain
[3]Biomedical Research Networking Center in Bioengineering, Biomaterials and Nanomedicine (CIBER-BBN), Barcelona, Spain
[4]ICREA, 08010 Barcelona, Spain

*Corresponding author. Email: elena.delcorro@icn2.cat

Supplementary Note 1: Materials and methods. Experimental details for graphene synthesis and device fabrication, morphological (Raman spectroscopy and AFM) and electrical characterization.

Raman Spectroscopy. Raman maps were acquired in a WITec spectrometer in backscattering configuration, with a 600 gr/nm grating and a 488 nm excitation wavelength. Power was kept below 2mW, to avoid sample damage, and focused using a 50x objective (Zeiss EC Epiplan 50x / 0.75) and a 63x immersion objective (Zeiss W Plan-Apochromat 63x / 1), for dry and electrolyte-immersed measurements, respectively. Raman maps were obtained over a 400 μm^2 area with 1 mm spatial resolution and for 3s acquisition time. The ionic concentration of the electrolyte, [KCl], was varied in a phosphate-buffered saline (PBS) solution at a fixed pH (7). Raman spectra were fitted to 2 Lorentzian contributions, corresponding to the G and 2D bands, at ~1580cm^{-1} and ~2700cm^{-1} respectively.

Atomic Force Microscopy (AFM). An MFP-3D Origin AFM (Asylum Research by Oxford Instruments) and a 150 Hz Al coated probe were used to characterize graphene surface in standard air-tapping mode. The Root Mean Square (RMS) roughness was calculated using a 25μm^2 region for each image.

Graphene macrotransistors fabrication and characterization. Graphene was grown on a 4x8 cm Copper foil (Alfa Aesar Graphene platform copper foil 99.95%, 0.035mm thickness) via Chemical Vapor Deposition (CVD), introducing H_2 and CH_4 flows at 1065ºC during 20 minutes at 25mbar.

Graphene macrotransistors design consisted of a 5x5 mm graphene active area, transferred on pyrex substrates using conventional PMMA and $FeCl_3$/HCl transfer process. Previously to the transfer, the substrates were contacted with Ti/Au (10/100nm) as metal contacts. Afterwards, devices were subjected to the standard and Cu-protection photolithography processes represented in Figure 4. Both strategies include the same fabrication steps: 1) Graphene definition (HiPR6512 photoresist, FujiFilm) and etched by reactive-ion etching (RIE) for 1

minute 2) Top metal definition (AZ5214E photoresist, Microchemicals) and 3) 2μm passivation layer (SU8-2005 photoresist, Kayaku Advanced Materials). However, for the Cu-protected samples, two additional steps were included: firstly, 20 nm of Cu were deposited prior to the graphene definition (e-beam deposition at a P < $5x10^{-7}$ mbar) and secondly, after the SU8-2005 passivation, the 20 nm Cu was etched using 0.02g/mL $(NH_4)_2S_2O_8$ for 7 minutes.

For the electrical characterization of the fabricated devices, current-voltage measurements were performed in solution-gate configuration (PBS 150 mM, pH 7) at a fixed Vds (50mV) and using Ag/AgCl as reference electrode.

Supplementary Note 2: Additional bibliographic review.

Table S1. Reported CNP of g-SGFETs. Single layer graphene is synthesized by CVD method on commercial copper foils and wet transferred onto Si/SiO_2 substrates using PMMA as supportive sacrificial polymeric layer. Transfer curve is measured at 100 mV Vds, using Ag/AgCl as reference electrode, in PBS solution (150 mM). Photolithography microfabrication steps were: 1. graphene definition / 2. top metal contacts / 3. Passivation.

Reference	Fabrication	CNP (V)
Levendorf et al. 2009 [1][a]	1-2	0.50
Hess et al. 2011 [2][b]	1-2-3	0.27 ± 0.02 (n=8)
Giacchetti et al. 2011 [3][c]	1-2-3	0.35
Kireev et al. 2017 [4]	1-2-3	0.35
Islam et al. 2020 [5][e]	1-2	0.07
Svetlova et al. 2021 [6][e]	1-2	0.40
Fomin et al. 2023 [7][d]	1-2	0.25
Schaefer et al. 2020 [8][c]	1-2-3	0.25
Brosel-Oliu et al. 2024 [9][c]	1-2-3	0.20 ± 0.01 (n=3)
Merino et al. 2024 [10][c]	1-2-3	0.39 ± 0.02 (n=2)

[a]Graphene is synthesized on evaporated Cu substrate
[b]Sapphire substrate
[c]Vds = 50 mV
[d] HPS solution (20mM HEPES, 150mM NaCl)
[e]CNP value is extracted from reported graph

Supplementary Note 3: Graphene charge carriers estimation by Raman spectroscopy

Raman spectro-electrochemistry of graphene transistors. Moving the gate voltage away from the Dirac point (V_g-V_{Dirac}) shifts the Fermi level (E_F) by electrostatically inducing charge carriers (n) according to Equation 1. Thanks to the strong electron-phonon coupling in graphene, the energy of the G phonon (ω_G) directly relates to the E_F, according to Equation 2[11], and therefore to n. (or its equivalent charge density, σ).

$$n = \frac{C_g\,(V_g - V_{Dirac})}{e} \qquad \text{Equation 1}$$

$$\hbar\omega_G - \hbar\omega_G^0 = \lambda\left(|E_F| + \frac{\hbar\omega_G}{4}\ln\frac{2|E_F| - \hbar\omega_G}{2|E_F| + \hbar\omega_G}\right) \qquad \text{Equation 2}$$

To keep the charge balance between graphene and its environment, graphene´s residual charges are screened by the electrolyte ions. Accordingly, increasing ionic strength shifts E_F and, in turn, ω_G. Figure S1 presents the magnitude of the ω_G shift ($\Delta\omega_G$), related to σ, as a function of the ionic strength after each step of the photolithography fabrication process.

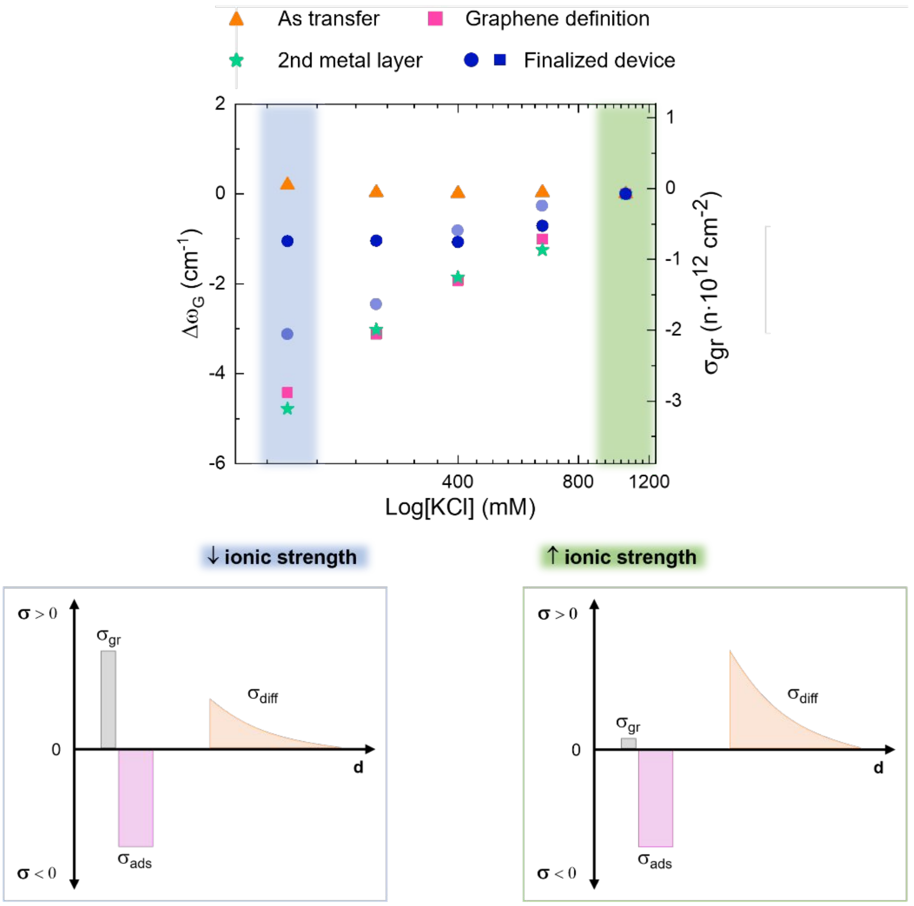

Figure S1. Adsorbed charges quantification by Raman spectroscopy. (a) Energy of the G phonon of graphene (and estimated adsorbed charge, σ_{gr}) as a function of the electrolyte ionic strength [KCl] at different fabrication stages. Each datum is an average from Raman maps of 400 μm^2 with 1 µm spatial resolution. Schematic representation of charge balance including graphene (σ_{gr}), residues (σ_{ads}) and the electrolyte (σ_{diff}), for low (b) and high (c) electrolyte ionic strength, [KCl]. To keep the equilibrium ($\sigma_{gr} + \sigma_{ads} + \sigma_{diff} = 0$), at high ionic strength the charge of the electrolyte screens the residual adsorbed charges.

Supplementary Note 4: Protection strategies review.

Table S2. Comparative of reported graphene-based device performance fabricated following different protective strategies (metal layers).

PROTECTION STRATEGY		DEVICE PERFORMANCE HIGHLIGHTS						
		AFM (rms/nm)		CNP (V)		Hole Mobility $cm^2/(V \cdot s)$		Configuration
Metal layer	Etchant	Standard	Protected	Standard	Protected	Standard	Protected	
Al (200 nm) [12] **	H3PO4+HNO3+ CH3COOH	2.52	0.52	Not reported	"Improved"	1159.00	1239.00	FET
Al (50nm) [13] **	BCl_3+Cl_2 (RIE)	3.04	1.90	Not reported	Not reported	762.93	1180.04	2-terminal
Al (20nm) [14] **	BCl_3+Cl_2 (RIE)	3.34	2.28	Not reported	Not reported	696.40	1103.10	2-terminal
Al (5nm) [15]	TMAH (diluted in developers)	1.00	0.20	Not reported	"Improved"	Not reported	~1500–3000	Top-gated FET Al_2O_3 (7.5nm)
Au (10nm) [16] *	Au etchant solution	Not mentioned	Not mentioned	37	19	~643	~1911	FET
Au (100nm) [17]	KI_3 solution	No AFM charact.		~0	Not reported	Not reported	122 (V_{TG}=0)<br>99 (V_{BG}=0)	Dual-gated FET Al_2O_3 (28nm)
Ti (2nm) [18]	DI:HF solution	No AFM charact.		2.48	7.09	~1142	~2235	Back-gated FET SiO_2 (90nm)
Ti (30nm) [19]	DI:HF solution	No AFM charact.		Not reported	Not reported	Not reported	Not reported	Back-gated FET
Y (5nm) [20]	DI:HCl solution	1.29	0.69	1.22	0.89	~1200	~3100	Back-gated FET SiO_2 (90nm)

*Au layer used either transfer and fabrication process

**Hall mobility reported

Supplementary Note 5: Cu protection additional characterization. Cu layer surface characterization by AFM and SEM. Cu etching evidence by XPS, CV and TOF-SIMS.

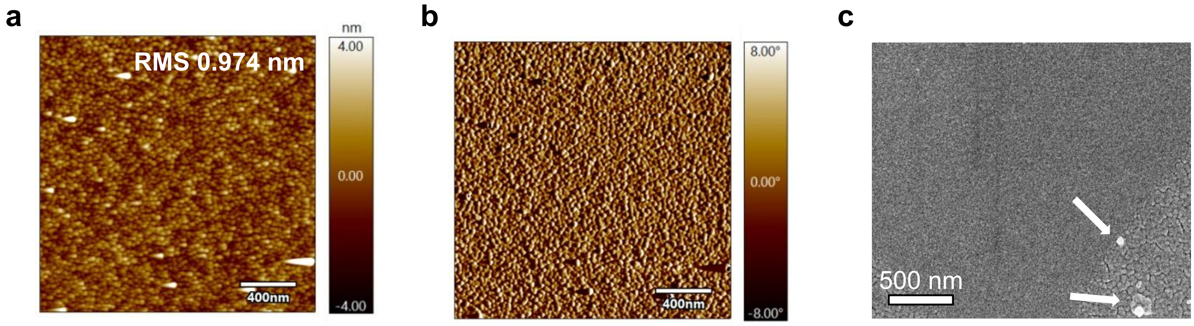

Figure S2. Protective Cu (20 nm) layer surface characterization. AFM characterization (scale bar 400 nm): **(a)** height image, corresponding RMS value, and **(b)** phase image. **(c)** Scanning electron microscopy (SEM) image acquired under high-vacuum conditions with a Quanta 650 FEG SEM and an Everhart-Thornley as a secondary electron signal detector. Electron voltage used was 2.0 kV with 10 mm working distance. Arrows point contamination particles around which copper layer does not close completely.

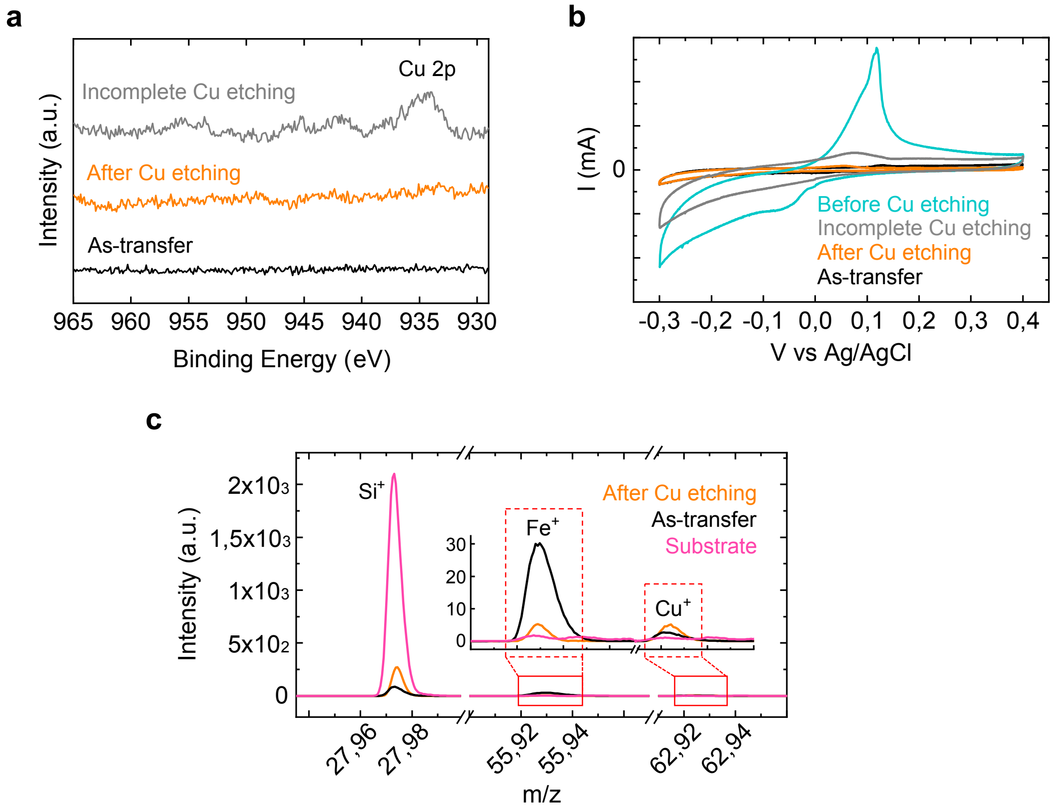

Figure S3. Cu etching chemical and electrochemical characterization.

(a) XPS spectra, Cu 2p region, for as-transfer (black) and Cu-processed devices after Cu etching in APS (0.02 g/mL) for 3 (grey) and 7 minutes (orange). XPS spectroscopy was performed in ultrahigh vacuum (5×10^{-10} mbar) with a SPECS PHOIBOS 150 hemispherical analyser using monochromatic Al Kα radiation with an energy of 1486.6 eV.

(b) Cyclic voltammetry (CV) of as-transfer (black) and Cu-processed devices before (cyan) and after Cu etching for 3 (grey) and 7 minutes (orange). CV was measured in a three-electrode configuration (graphene, Pt wire and Ag/AgCl as working, counter and reference electrode, respectively), in PBS solution (150 mM, pH 7), with a 20 mV/s scan rate, using a BioLogic SP-200 potentiostat.

(c) Time-of-Flight Secondary Ion Mass Spectrometry (ToF-SIMS) positive spectra, Si^+, Fe^+ and Cu^+ regions, for the Si/SiO_2 substrate (pink), the as-transfer device (black) and the Cu-processed device after complete Cu etching (orange). For Cu-processed devices after Cu etching the Cu amount is comparable with the as-transfer graphene device. Surface analysis was performed by EUROFINS Scientific using an Ion-Tof TOF-SIMS IV instrument operated in both positive and negative mode with 25keV Bi^+ primary ions. For each sample, 3 areas of 500x500 μm^2 were measured and averaged.

Supplementary Note 6: Electrical characterization. Comparison and CNP statistics for standard and Cu-protected fabricated macrotransistors.

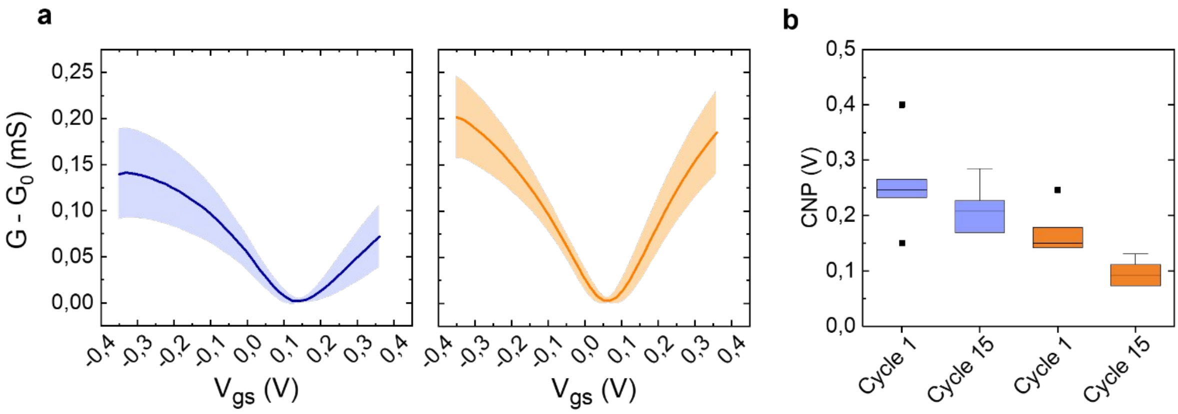

Figure S4. Graphene macrotransistors electrical characterization. Statistical (n=4) transistor transfer curve for (a) standard processed and (b) Cu-processed devices. (c) Comparative CNP analysis (n=7).

Supplementary references

(1) Levendorf, M. P.; Ruiz-Vargas, C. S.; Garg, S.; Park, J. Transfer-Free Batch Fabrication of Single Layer Graphene Transistors. *Nano Lett.* **2009**, *9* (12), 4479–4483. https://doi.org/10.1021/nl902790r.

(2) Hess, L. H.; Hauf, M. V.; Seifert, M.; Speck, F.; Seyller, T.; Stutzmann, M.; Sharp, I. D.; Garrido, J. A. High-Transconductance Graphene Solution-Gated Field Effect Transistors. *Appl. Phys. Lett.* **2011**, *99* (3), 033503. https://doi.org/10.1063/1.3614445.

(3) Giacchetti, B. M.; Hsu, A.; Wang, H.; Kim, K. K.; Kong, J.; Palacios, T. CVD-Grown Graphene Solution-Gated Field Effect Transistors for pH Sensing. *MRS Proc.* **2011**, *1283*, mrsf10-1283-b03-07. https://doi.org/10.1557/opl.2011.583.

(4) Kireev, D.; Brambach, M.; Seyock, S.; Maybeck, V.; Fu, W.; Wolfrum, B.; Offenhäusser, A. Graphene Transistors for Interfacing with Cells: Towards a Deeper Understanding of Liquid Gating and Sensitivity. *Sci. Rep.* **2017**, *7* (1), 6658. https://doi.org/10.1038/s41598-017-06906-5.

(5) 2020_Graphene-Based Electrolyte-Gated Field-Effect Transistors forPotentiometrically Sensing Neuropeptide Y in PhysiologicallyRelevant Environments_Appl Nano Mater_Ahmad E Islam et al.Pdf.

(6) Svetlova, A.; Kireev, D.; Mayer, D.; Offenhäusser, A. Origins of Leakage Currents on Electrolyte-Gated Graphene Field-Effect Transistors.

(7) Fomin, M.; Jorde, L.; Steinbach, F.; You, C.; Meyer, C. Liquid-Gated Graphene Field-Effect Transistors for Biosensing on Lipid Monolayers. *Phys. Status Solidi B* **2023**, *260* (12), 2300324. https://doi.org/10.1002/pssb.202300324.

(8) Schaefer, N.; Garcia-Cortadella, R.; Calia, A. B.; Mavredakis, N.; Illa, X.; Masvidal-Codina, E.; Cruz, J. D. L.; Corro, E. D.; Rodríguez, L.; Prats-Alfonso, E.; Bousquet, J.; Martínez-Aguilar, J.; Pérez-Marín, A. P.; Hébert, C.; Villa, R.; Jiménez, D.; Guimerà-Brunet, A.; Garrido, J. A. Improved Metal-Graphene Contacts for Low-Noise, High-Density Microtransistor Arrays for Neural Sensing. *Carbon* **2020**, *161*, 647–655. https://doi.org/10.1016/j.carbon.2020.01.066.

(9) 2024_Single-Step Functionalization Strategy of Graphene Microtransistor Array with Chemically Modified Aptamers for Biosensing Applications_Small_S. Brosel-Oliu et al.Pdf.

(10) Merino, J. P.; Brosel-Oliu, S.; Rius, G.; Illa, X.; Sulleiro, M. V.; Del Corro, E.; Masvidal-Codina, E.; Bonaccini Calia, A.; Garrido, J. A.; Villa, R.; Guimerà-Brunet, A.; Prato, M.; Criado, A.; Prats-Alfonso, E. Ethanol Solvation of Polymer Residues in Graphene Solution-Gated Field Effect Transistors. *ACS Sustain. Chem. Eng.* **2024**, *12* (24), 9133–9143. https://doi.org/10.1021/acssuschemeng.4c01538.

(11) Yan, J.; Zhang, Y.; Kim, P.; Pinczuk, A. Electric Field Effect Tuning of Electron-Phonon Coupling in Graphene. *Phys. Rev. Lett.* **2007**, *98* (16), 166802. https://doi.org/10.1103/PhysRevLett.98.166802.

(12) Chen, Z.; Li, Y.; Wang, B.; Wei, B.; Yang, L. Enhanced Photolithography with Al Film Insertion for Large-Scale Patterning of CVD Graphene. *Opt. Mater. Express* **2018**, *8* (8), 2403. https://doi.org/10.1364/OME.8.002403.

(13) Wang, Y.; Su, N.; Wei, S.; Wang, J.; Li, M. Enhancing the Consistency and Performance of Graphene-Based Devices via Al Intermediate-Layer-Assisted Transfer and Patterning. *Nanomaterials* **2024**, *14* (7), 568. https://doi.org/10.3390/nano14070568.

(14) Wang, J.; Wang, Y.; Su, N.; Li, M. Improving Consistency and Performance of Graphene-Based Devices via Al Sacrificial Layer. *Colloid Interface Sci. Commun.* **2023**, *56*, 100743. https://doi.org/10.1016/j.colcom.2023.100743.

(15) Hsu, A.; Wang, H.; Kim, K. K.; Kong, J.; Palacios, T. Impact of Graphene Interface Quality on Contact Resistance and RF Device Performance. *IEEE Electron Device Lett.* **2011**, *32* (8), 1008–1010. https://doi.org/10.1109/LED.2011.2155024.

(16) Choi, W. J.; Chung, Y. J.; Park, S.; Yang, C.; Lee, Y. K.; An, K.; Lee, Y.; Lee, J. A Simple Method for Cleaning Graphene Surfaces with an Electrostatic Force. *Adv. Mater.* **2014**, *26* (4), 637–644. https://doi.org/10.1002/adma.201303199.

(17) Ahlberg, P.; Hinnemo, M.; Song, M.; Gao, X.; Olsson, J.; Zhang, S.-L.; Zhang, Z.-B. A Two-in-One Process for Reliable Graphene Transistors Processed with Photo-Lithography. *Appl. Phys. Lett.* **2015**, *107* (20), 203104. https://doi.org/10.1063/1.4935985.

(18) Joiner, C. A.; Roy, T.; Hesabi, Z. R.; Chakrabarti, B.; Vogel, E. M. Cleaning Graphene with a Titanium Sacrificial Layer. *Appl. Phys. Lett.* **2014**, *104* (22), 223109. https://doi.org/10.1063/1.4881886.

(19) Theofanopoulos, P. C.; Ageno, S.; Guo, Y.; Kale, S.; Wang, Q. H.; Trichopoulos, G. C. High-Yield Fabrication Method for High-Frequency Graphene Devices Using Titanium Sacrificial Layers. *J. Vac. Sci. Technol. B Nanotechnol. Microelectron. Mater. Process. Meas. Phenom.* **2019**, *37* (4), 041801. https://doi.org/10.1116/1.5098324.

(20) Wang, N. C.; Carrion, E. A.; Tung, M. C.; Pop, E. Reducing Graphene Device Variability with Yttrium Sacrificial Layers. *Appl. Phys. Lett.* **2017**, *110* (22), 223106. https://doi.org/10.1063/1.4984090.
